# Oxygen Supplementation to Stabilize Preterm Infants in the Fetal to Neonatal Transition: No Satisfactory Answer

**DOI:** 10.3389/fped.2016.00029

**Published:** 2016-04-19

**Authors:** Isabel Torres-Cuevas, Maria Cernada, Antonio Nuñez, Javier Escobar, Julia Kuligowski, Consuelo Chafer-Pericas, Maximo Vento

**Affiliations:** ^1^Neonatal Research Group, Health Research Institute La Fe, Valencia, Spain; ^2^Division of Neonatology, University and Polytechnic Hospital La Fe, Valencia, Spain; ^3^Spanish Maternal, Infant and Developmental Network (Red SAMID), Spanish Ministry of Economy and Competitiveness, Madrid, Spain

**Keywords:** prematurity, oxygen, oxidative stress, free radicals, biomarkers, oxygen saturation, pulse oximetry

## Abstract

Fetal life elapses in a relatively low oxygen environment. Immediately after birth with the initiation of breathing, the lung expands and oxygen availability to tissue rises by twofold, generating a physiologic oxidative stress. However, both lung anatomy and function and the antioxidant defense system do not mature until late in gestation, and therefore, very preterm infants often need respiratory support and oxygen supplementation in the delivery room to achieve postnatal stabilization. Notably, interventions in the first minutes of life can have long-lasting consequences. Recent trials have aimed to assess what initial inspiratory fraction of oxygen and what oxygen targets during this transitional period are best for extremely preterm infants based on the available nomogram. However, oxygen saturation nomogram informs only of term and late preterm infants but not on extremely preterm infants. Therefore, the solution to this conundrum may still have to wait before a satisfactory answer is available.

## Introduction

Good and evil we know in the field of this world grow up together almost inseparably.

Paradise Lost (John Milton; 1608–1674)

Adaptation to extra uterine life implies major cardiovascular and respiratory changes that occur in a timely fashion along the first few minutes after birth. Placental gas exchange is substituted by air-borne lung respiration. The initial breathing movements yield an increment of the oxygen content in arterial blood. Hence, partial pressure of oxygen (p_a_O_2_) increases from 3.3 kPa (25–30 mmHg) in the fetus to 10.5 kPa (75–85 mmHg) in the newly born only 5 min after cord blood circulation ceases. Increased p_a_O_2_ elicits the dilatation of the pulmonary vascular bed, reduction of pulmonary vascular resistance, enhances pulmonary blood flow, left heart preload, and closure of intra- and extracardiac shunts (1). An adequate oxygenation is indispensable not only for providing the energy required for the normal functionality of oxy-regulator tissues, such as heart and brain, but also for growth and development of the newborn infant.

This extraordinary ensemble of timely and synchronized physiological responses requires the maturity of the cardiovascular, the respiratory, and the antioxidant (AO) defense systems.

The present review will focus on the fundamental physiologic role of oxygen during the fetal to neonatal transition and the difficulties to translate our yet imprecise and incomplete knowledge of oxygen metabolism into clinical protocols that regulate oxygen supplementation, especially to preterm infants, to avoid the deleterious consequences derived from a lack or an excess of oxygen.

## Oxygen and Oxidative Stress

### Aerobic Metabolism and Oxygen Physiology

**Figure 1:**
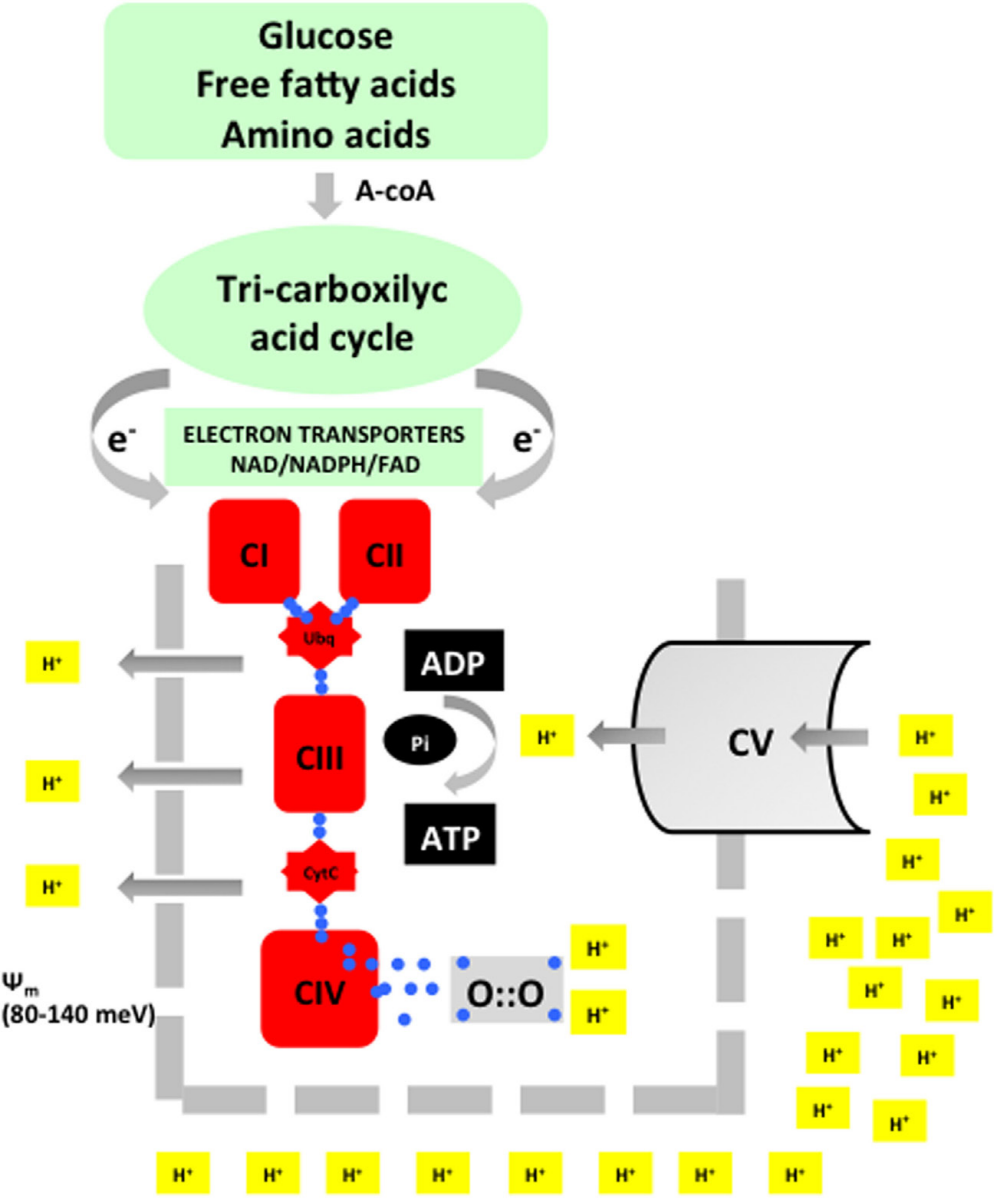
**Acetyl-coA is the merging metabolite derived from basic nutrients**. Entering into the inner mitochondrial space acetyl-coA will undergo a metabolic transformation in the tricarboxylic cycle (Kreb’s cycle). During this process, highly energized electrons are liberated and transported by specific electron transporters to the electron transport chain (ETC). Energy is used to extrude protons and thus establish a transmembrane potential (Ψ_m_). In the final step, ATP synthase will again intrude protons in the inner mitochondrial space. The energy provided by Ψ_m_ will be employed to synthesize adenosine triphosphate (ATP) from adenosin diphosphate (ADP). Oxygen will be reduced with four electrons and combined with two protons to form water. This process is known as oxidative phosphorylation.

Oxygen (O_2_) is indispensable for the provision of sufficient energy to allow multicellular organisms an adequate growth and development. Nutritional components, such as carbohydrates, fat, and proteins, are converted during digestion in simple molecules, such as glucose, fatty acids, or amino acids. Combustion of these nutritional elements in the mitochondria in the presence of oxygen occurs in a two-step process known as oxidative phosphorylation (Figure 1). The energy balance derived from oxidative phosphorylation is substantially more efficient than in anaerobic metabolism. Hence, while 1 mol of glucose generates a positive balance of 2 mol of adenosine triphosphate (ATP) under anaerobic conditions, in the presence of O_2_, it will generate 32–36 mol of ATP. Thus, the energy efficiency of aerobic is 16–18 times that of anaerobic metabolism (2).

**Figure 2:**
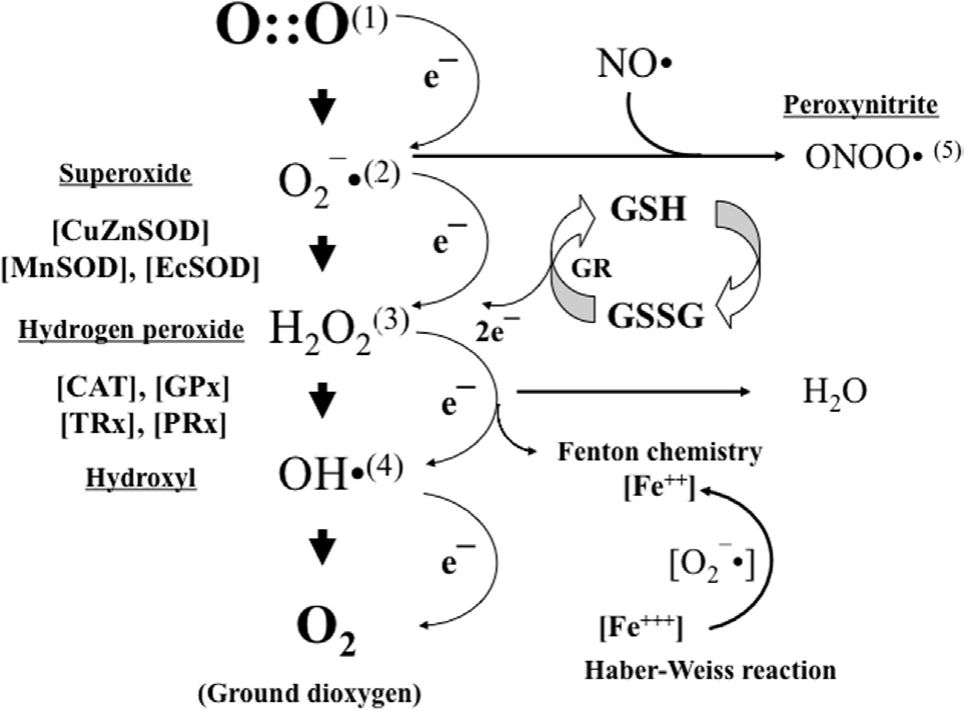
**Oxygen (1) is stepwise reduced by just one electron leading to the formation of anion superoxide (2)**. Anion superoxide is dismutated by superoxide dismutases (SODs) to hydrogen peroxide (3), which in turn is transformed into water and oxygen by the action of catalases (CATs) and glutathione peroxidase (GPX). In the presence of transition metals (e.g., iron and copper), hydrogen peroxide can be transformed into hydroxyl radical (4). Moreover, in the presence of nitric oxide (NO), anion superoxide can also be transformed into peroxynitrite (5). Anion superoxide, hydroxyl radical, and peroxynitrite are highly reactive free radicals that will cause structural and functional damage to nearby standing molecules. Hydrogen peroxide will act as a cell-signaling molecule. Reduced glutathione (GSH) is the most relevant non-enzymatic antioxidant in the cell cytoplasm and an essential determinant of cell’s redox balance.

O_2_ is present in nature generally as ground molecular di-oxygen. To achieve conformational stability, O_2_ needs to be reduced by four electrons. Paramagnetic properties confer O_2_ with a low reactivity, and therefore, to establish bonds with other compounds oxygen, it will undergo a step-by-step tetravalent reduction. However, incomplete reduction with just one electron at a time will lead to the formation of intermediate reactive oxygen species (ROS), such as anion superoxide (O^2−^), hydrogen peroxide (H_2_O_2_), or hydroxyl radical (OH^⋅^) (Figure 2).

Superoxide and hydroxyl radical both are free radicals. Free radicals are atomic or molecular species capable of independent existence that contain one or more unpaired electrons in their molecular orbitals. They are able, therefore, to oxidize cellular membranes, structural and functional proteins, and nucleic acids. In the presence of nitric oxide (^⋅^NO), oxygen-derived free radicals will react forming reactive nitrogen species (RNS) such as peroxynitrite (ONOO^−^). ROS and RNS are potent oxidizing and reducing agents that subtract electrons from any nearby standing molecules altering their structure and function, and begetting them into free radicals (2–4).

In recent years, the concept of the redox code has been postulated. The redox code consists of four principles by which biological systems are organized being O_2_ central to all of them. These include electron donating and accepting properties of nicotinamide adenine dinucleotide (NAD) and NAD phosphate (NADP) linked to ATP production and catabolism; protein tertiary structure conformation through kinetically controlled redox switches that regulate interactions, trafficking activity, etc.; the role of hydrogen peroxide in redox-sensing mechanisms; and finally the ability of redox networks to conform to an adaptive system that responds to the environment (5). Interestingly, the response to the environment is performed through subcellular systems from microcompartments (cytoplasm, endoplasmic reticulum, nucleus, and extracellular space) that have an individual and characteristic redox potential that can be altered individually without a concomitant alteration of the other tissue territories. As a consequence, the older concept of oxidative stress as a global situation affecting the entire economy is under review (6, 7).

### Antioxidant Defenses

To overcome the deleterious actions of oxygen free radicals, AO defense systems have evolved (Figure 2). AO defenses can be enzymatic and non-enzymatic in nature. The families of superoxide dismutase (SOD), catalase (CAT), glutathione peroxidase (GPX), and glucose 6-phosphate dehydrogenase (G6PD) represent, from a clinical perspective, the most relevant AO enzymes. In addition, there are a series of compounds capable of neutralizing ROS and free radicals. The most abundant cytoplasmic non-enzymatic AO is glutathione (GSH), a ubiquitous tripeptide formed by γ-glutamine, l-cysteine, and glycine. GSH easily combines with another GSH resulting in GSSG (oxidized glutathione), thus providing two electrons, which are used to satisfy the promiscuous appetite of free radicals. GSSG is reduced again to GSH by glutathione reductase (GSH-reductase) with the electrons coming from the Krebs cycle and provided by NADPH (reduced form of NADP) (Figure 2). Other relevant non-enzymatic AO are proteins that bind transition metals, such as transferrin and ceruloplasmin, or molecules that quench free radicals, such as uric acid and bilirubin, and certain vitamins, such as A, E, and C (2).

Oxidative stress refers to the disequilibrium that ensues in an organism when the formation of free radicals overrides the ability of biologic system to completely neutralize them. Oxidative stress can be physiologic in nature (oxidative eustress) and necessary to activate specific metabolic pathways or pathologic (oxidative distress), which causes damage to structures and alters the function of biological systems. Oxidative stress can result from diminished AO levels or AO enzymes’ response capacity, or defects in the genes that regulate the AO machinery. In addition, oxidative stress may also be the consequence of an increased supplementation with O_2_, toxic substances, radiation, etc. (2–4).

Enzymatic and non-enzymatic AO defenses mature late in gestation. Therefore, preterm infants, especially those who are very preterm (≤28 weeks GA), are endowed with an immature AO defense system that is characterized by a diminished capacity of response to a pro-oxidant aggression (8). In addition, preterm infants are depleted of non-enzymatic AO, such as vitamins (E, D, C, flavonoids, and carotenoids), or micronutrients (selenium, copper, zinc, etc.) that accumulate in the fetus at the end of gestation (9).

Biomarkers employed to evaluate oxidative stress may directly reflect a pro- or anti-oxidant status (redox status) such as GSH/GSSG ratio. Furthermore, the may inform on damage to the cell components, such as lipids (malondialdehyde or *n*-aldehydes), nucleic acids [8-oxo-deyhydroguanosine (8-oxodG)], or proteins (oxidized tyrosine and carbonyl compounds) (2). In recent years, isoprostanes and isofurans have evolved as one of the most reliable markers of oxidative stress assessing peroxidation of poly-unsaturated fatty acids (PUFA) (Table 1) (10, 11).

**Table 1:** **Oxidative stress and damage biomarkers used in the clinical setting and in human research, targeted biofluids, and recommended analytical techniques**.

Oxidative biomarkers	Target biomolecule	Modification	Biological sampling	Analytical method
Glutathione (GSH/GSSG ratio)	Antioxidants	General redox status	Total blood	LC–MS/MS
MDA	Lipids	PUFA peroxidation	Plasma	HPLC (UV detection)
HNE	Lipids	PUFA peroxidation	Plasma	HPLC
o-Tyrosine (o-Tyr/Phe ratio)	Proteins	Tyrosine hydroxylation	Urine	LC–MS/MS
m-Tyrosine (m-Tyr/Phe ratio)	Proteins	Tyrosine hydroxylation	Urine	LC–MS/MS
3N2-tyrosine	Proteins	Tyrosine nitratation	Urine	LC–MS/MS
8OHdG (8OHdG/2dG ratio)	Lipids	AA peroxidation	Urine/plasma	LC–MS/MS
F2-IsoPs	Lipids	AA peroxidation	Urine/plasma	GC–MS/MS; LC–MS/MS
D2/F2-ISoPs	Lipids	AA peroxidation	Urine/plasma	GC–MS/MS; LC–MS/MS
IsoFs	Lipids	AA peroxidation	Urine/plasma	GC–MS/MS; LC–MS/MS
NeuPs	Lipids	DHA peroxidation	Urine/plasma	GC–MS/MS; LC–MS/MS
NeuFs	Lipids	DHA peroxidation	Urine/plasma	GC–MS/MS; LC–MS/MS

*Modified from Vento et al. (2)*.

*GSH, reduced glutathione; GSSG, oxidized glutathione; MDA, malondialdehyde; HNE, 4-hydrox-2-non-enal; o-Tyr, ortho-tyrosine; m-Tyr, meta-tyrosine; 3N2-Tyrosine, 3-nitrotyrosine; 8OHdG, 8-hydroxi-2′-deoxiguanosine; 2dG, 2′-deoxiguanosine; IsoPs, isoprostanes; IsoFs, isofurans; NeuPs, neuroprostanes; NeuFs, neurofurans; AA, arachidonic acid; DHA, docosahexaenoic acid; LC, liquid chromatography; GC, gas chromatography; MS/MS, tandem mass spectrometry*.

Reactive oxygen species and RNS also trigger proinflammatory responses in the cells promoting the activation of nuclear factor-kappa B (NF-κB), a transcription factor for multiple inflammation-related genes, and tumor necrosis factor alpha (TNFα), crucial in the inflammatory response as well as in the activation of apoptosis (12). ROS act upon redox mechanisms, which control gene expression, cell proliferation, and apoptosis. Diffusible H_2_O_2_ especially, but also other ROS, act reversibly oxidizing and reducing signaling proteins, providing a means for control of protein activity, protein–protein interaction, protein trafficking, and protein–DNA interaction (5, 9).

### Maturation of the Antioxidant System

In experimental studies, it has been documented that AO enzyme levels in the lung of different mammals are not expressed until late in gestation. The timing of development of both fetal lung AO enzymes (SOD, CAT, and GPX) and surfactant followed a similar pattern of prenatal biochemical maturation. Hence, during the final 10–15% of gestation, there was an exponential increase of 150–200% activity in the AO enzymes present in lung tissue. Moreover, simultaneously, there was an essentially parallel rapid rise in lung surfactant content. Both these developmental changes suggest a preparation to facilitate the establishment of an effective alveolar recruitment and gas exchange after birth (13, 14). Consequently, preterm infants with an immature AO enzyme system will be predisposed to oxidative stress-associated lung damage. Notably, the use of antenatal steroids increases the AO enzyme activity, especially in preterm females improving their ability to satisfactorily transit into the extra uterine world (8).

Resistance to oxidative stress also relies on non-enzymatic AOs, such as vitamins (A, E, and C), and compounds, such as glutathione, peroxiredoxin, or thioredoxin. Significantly higher levels of vitamins A, E, and C have been found in term as compared to preterm infants (9). However, supplementation with ascorbic acid and tocopherol to baboons submitted to a hyperoxia did not effectively offset oxidative stress-derived lung damage (15).

The GSH/GSSG ratio is considered one of the most reliable markers of cellular reducing state (16). Notably, the physiologic oxidative stress inherent to fetal to neonatal transition favors the upregulation of specific enzymes, such as γ-cystathionase in the trans-sulfuration pathway, which is the limiting enzyme that facilitates l-cysteine synthesis and thus glutathione synthesis (17). Remarkably, γ-cystathionase is also expressed late in gestation, and therefore, l-cysteine renders an indispensable amino acid in very preterm infants. Moreover, synthesis of GSH below 32 weeks gestation is significantly lower than in late preterm or term infants predisposing these babies to an increased oxidative stress in the fetal to neonatal transition (18).

## Oxygen in Fetal Life

### Fetal Oxygen Environment

Fetal life develops in an environment relatively hypoxic, when compared to the extra uterine; hence, *in utero* p_a_O_2_ is ≈3.5–4.5 kPa (25–35 mmHg) while in adults is ≈11.5–13.0 kPa (80–90 mmHg) (1). However, in spite of this, fetal tissues receive an oxygen load that does not substantially differ from the newborn or adult. The presence of fetal hemoglobin with a greater affinity for oxygen enhances oxygen uptake from the intervillous space by fetal erythrocytes. As a consequence, fetuses have increased oxygen saturation (SpO_2_) for a given p_a_O_2_ as compared to adults. Furthermore, the fetal cardiac output is extremely high reaching values of 250–300 mL/kg/min, thus providing tissue with an adequate flow rate in spite of lower oxygen content. Moreover, venous return from the placenta is directed to the most oxygen demanding organs, such as heart and central nervous system, through extra- and intracardiac shunting (19).

**Figure 3:**
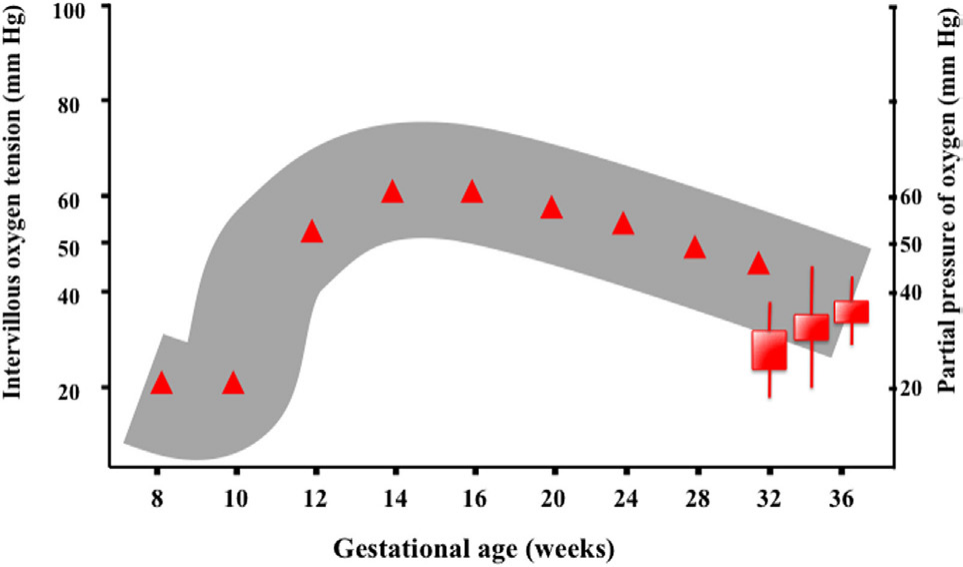
**Oxygenation of the fetus is clearly dependent on the partial pressure gradients between maternal blood, placental tissue, fetal blood, and tissue**. The level of intervillous oxygen concentration varies along gestation. Studies performed in human fetuses have shown that before the 12th week of gestation, the intervillous partial pressure of oxygen (PIVO_2_) has a median value around 18–20 mmHg, presumably to protect the embryo, which is highly sensitive to ROS. However, PIVO_2_ rises in the following weeks reaching a peak value (60 mmHg) around 14–16 weeks of gestation. Thenceforth, PIVO_2_ decreases slowly, reaching values of 45–48 mmHg at 36 weeks of gestation (9).

Oxygenation of the fetus is clearly dependent on the partial pressure gradients between maternal blood, placental tissue, fetal blood, and tissue. Remarkably, the level of placental oxygen, as measured in the intervillous space, differs at different stages of gestation (Figure 3). The provision of an adequate amount of oxygen to the fetus shifts along gestation. Hence, levels considered normal early in pregnancy (8–14 weeks gestation) would be indicative of hypoxia in late pregnancy (>24 weeks gestation). Noteworthy, during the embryonic stage of development, the fetus is extremely sensitive to oxidative stress, and an environment of low oxygen is required for an adequate development. In the second half of pregnancy, the placenta will provide the fetus with higher amounts of oxygen and nutrients, allowing for an exponential increase in size and weight. Hence, studies performed in human fetuses have shown that before the 12th week of gestation, the intervillous partial pressure of oxygen has a median value around 18–20 mmHg but rises steeply peaking at 60 mmHg around 14–16 weeks gestation, and thereafter decreases slowly reaching values of 45–48 mmHg at 36 weeks of gestation (20, 21).

### Fetal Oxygen Sensing

**Figure 4:**
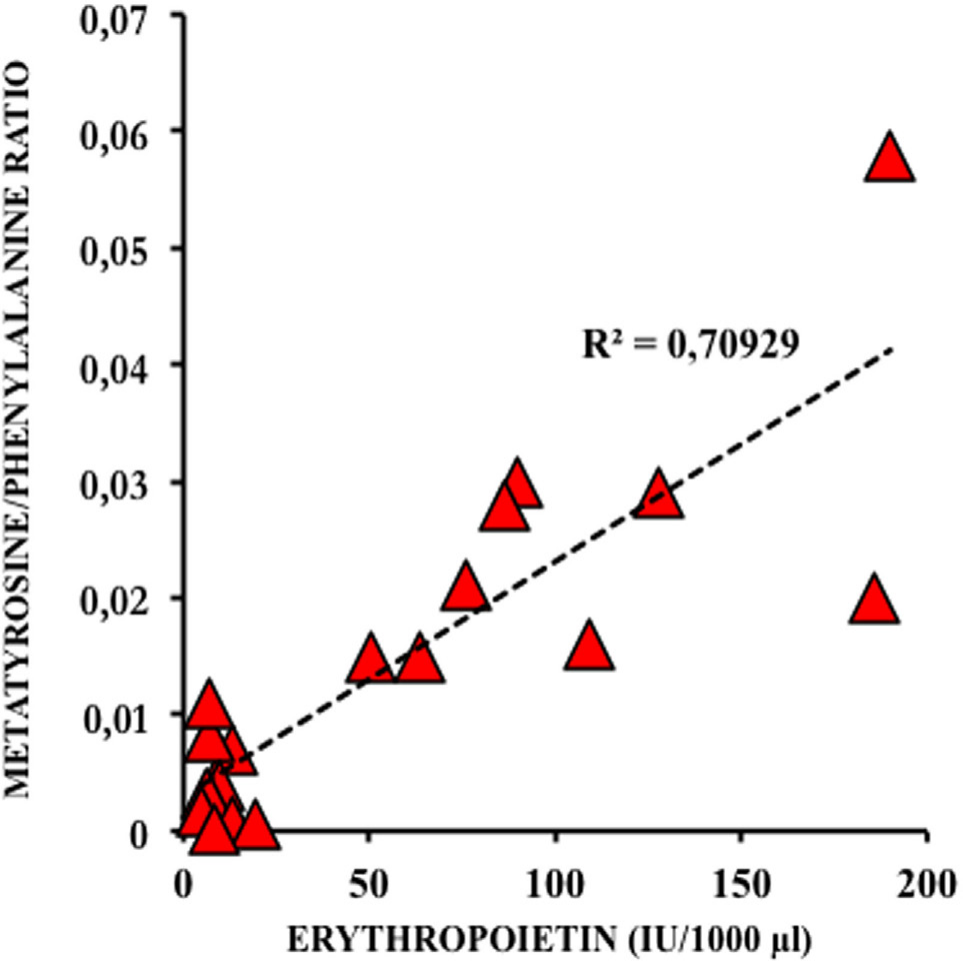
**Correlation between amniotic fluid levels of erythropoietin (EPO) and meta-tyrosine, a biomarker of oxidative damage to protein in diabetic type 2 pregnancies treated with insulin (23)**.

Physiological systems are adapted to sense O_2_ needs not only to avoid excessive exposures to ROS but also to provide sufficient O_2_ to allow normal cellular functioning. The most relevant oxygen sensing transcription factors are hypoxia-inducible factor (HIF), cAMP response-binding protein (CREB), nuclear factor kappa B (NFκB), activator protein 1 (AP-1), and p53. These factors regulate gene expression in response to changes in the concentration of oxygen and derived ROS. Low O_2_ containing environments favor early placental and fetal development. Angiogenesis is stimulated by low oxygen tissue concentrations through transcriptional and post-transcriptional regulation of growth factors, such as vascular endothelial growth factors (VEGFs), erythropoietin (EPO), placental growth factor (PGF), and angiopoietins 1 and 2. The master regulator for the cell’s adaptive responses to hypoxia is HIF-1, a heterodimeric transcription factor comprising HIF-1α and HIF-1β subunits (22). Activated genes, especially VEGF and EPO, enhance O_2_ delivery to tissue. EPO does not cross the placenta, and it does not accumulate in tissue. Therefore, fetal plasma concentration of EPO is determined by its rate of synthesis and elimination, and amniotic fluid EPO levels correlate well to fetal and neonatal plasma EPO levels obtained simultaneously at elective and emergency cesarean section. Amniotic fluid EPO concentration increases exponentially during fetal hypoxia and correlates to oxidative and nitrosative stress in the fetus (Figure 4). Alteration of EPO concentration in amniotic fluid correlates to immediate postnatal complications and could, therefore, be used as a reliable biomarker to indicate emergency C section (23).

## Fetal to Neonatal Transition and Postnatal Adaptation

### Oxidative Stress in the Fetal to Neonatal Transition

Even with a mature AO system, the burden of ROS as a consequence of the fetal to neonatal transition renders oxidative stress in the immediate postnatal period inevitable even breathing 21% oxygen. In an experimental study at different stages of development in rats, GSH/GSSG ratio decreased 15- to 20-fold in isolated hepatocytes from fetal to neonatal and to adult animals due an exponential increase in GSSG indicating that a pro-oxidant status is generated in the fetal to neonatal transition (24). Gelfand et al. showed that the use of pure oxygen after a hypoxic episode in intubated and ventilated rat pups was able to significantly reduce the GSH/GSSG ratio causing an intense oxidative stress (25). In addition, Presti et al. showed short- and long-term outcome differences between room air and oxygen-resuscitated subjects were demonstrated in a P7/8 murine model (26). In pups resuscitated with pure oxygen, cerebral blood flow was more rapidly restored, however, with worsened short-term sensorimotor deficit raising additional concerns regarding post-resuscitation hyperemia (26). Notably, in a very recent experimental study with an innovative design, offspring of pregnant mice were allowed to deliver in a low oxygen atmosphere mimicking *in utero* milieu (FiO_2_ = 0.14). Pups were kept at this low FiO_2_ for 8 h. Biochemical analysis of lung tissue revealed an increased GSH/GSSG ratio in lung in day 1 and in the brain in day 7 after birth as compared to pups born in room air (FiO_2_ = 0.21). Interestingly, mRNA expression of NRF-2-related AO genes was significantly increased in lung and brain (27). Apparently, a smooth transition from the lower oxygen milieu *in utero* to the relatively hyperoxic milieu *ex utero* seemed to be protective. Translating these findings into the clinical setting would imply avoiding targeting high saturations too rapidly in preterm infants after birth.

The use of high concentrations of oxygen even for short periods of time has deleterious effects on the lung. Hence, free radicals cause an inflammatory response with destruction of the alveolar–capillary barrier, increased pulmonary permeability, and endothelial and epithelial cell death, leading to citoarchitectural alterations and predisposition to chronic lung disease [for a review, see Ref. (28)]. Moreover, stabilization with high oxygen concentrations is not only toxic to the lungs but also to different organs such as heart, liver, and brain. Studies in a hypoxic piglet model of hypoxia-reoxygenation showed that the use of pure oxygen caused not only an increased concentration of extracellular glycerol in the brain striatum but also increased matrix metalloproteinases in lung, liver, heart, and brain compared with the use of room air. Interestingly, there is clear dose-dependent oxygen toxicity, and elimination of biomarkers of oxidative damage caused to protein and DNA significantly correlated to the FiO_2_ provided during resuscitation (29).

### Circulatory Changes in the Fetal to Neonatal Transition

During fetal life, the gas exchange between mother and fetus is performed across the placenta. In the fetal circulation, most of the right ventricular output is directed through the ductus arteriosus to the aorta, and only 5–10% of the combined ventricular output is directed to the pulmonary vascular bed. Pulmonary vascular resistance increases with gestational age reaching values equivalent to systemic pressure. Vascular tone of the fetal lung is maintained by different pathways, which include high vasoconstriction agents, such as low oxygen tension, endothelin-1, leukotrienes, and Rho kinase, and low basal level of vasodilators, such as prostacyclin and nitric oxide (NO) (30). Abrupt changes in the pulmonary circulation occur immediately after cord clamping and the initiation of the first breaths. These are characterized by a rapid fall in the pulmonary vascular resistance and pulmonary artery pressure, and a 10-fold rise in pulmonary blood flow, which becomes the sole recipient of right ventricular output. Moreover, pulmonary blood flow represents few minutes after birth, the only source of preload for the left ventricle. The most critical signals for these transitional changes are mechanical distension of the lung, a decrease in carbon dioxide tension, and an increase in oxygen tension in the lungs. Altogether, these changes are triggered by the elimination of the fluid filling the lungs and subsequent lung aeration (31). In term infants, it takes only about few minutes to almost completely aerate the lung. It has been generally accepted that the principal mechanism by which lung liquid is cleared from the airways at birth is *via* adrenaline-induced sodium reabsorption, which reverses the osmotic gradient that drives liquid secretion across the pulmonary epithelium during fetal life. However, recent studies using phase contrast X-ray imaging techniques have shown that the transpulmonary hydrostatic pressure gradient generated with the first inspiratory movements provides the driving force that clears lung fluid into the interstitial tissue, where it is rapidly eliminated through the lymphatic or the macrophages. Remarkably, no liquid clearance from the airways has been detected during the expiratory phase. However, it is possible that transepithelial Na^+^ reabsorption plays a critical role in retaining liquid within the interstitial tissue compartment, thereby preventing its re-entry into the alveolar space during expiration (32). These findings have a direct translational application. Hence, a positive pressure in the airway will decisively contribute to a rapid aeration of the lung and an efficient gas exchange. This can be elicited by a spontaneously breathing pattern in the health newborn or will need to be achieved, employing an adequate ventilation strategy by the resuscitation team that includes positive-end expiratory pressure. However, the use of a sustained inflation, although successful in experimental studies (33), has not shown to be provide substantial advantages for preterm infants in the clinical setting. In a randomized controlled multicenter trial performed in Italy, preterm infants <29 weeks gestation were administered an initial sustained lung inflation of 25 cmH_2_O for 15 s followed by nCPAP or nCPAP alone. The primary outcome, which was the need for mechanical ventilation in the first 72 h after birth, was significantly lower in the sustained inflation group. However, no differences regarding the need for respiratory support or survival without BPD were shown. Moreover, an increase in the incidence of pneumothorax was present in the sustained lung inflation group (34). Notwithstanding, an ongoing multicenter international randomized controlled trial adequately powered, which includes spontaneously breathing preterm babies of 23–26 weeks gestation receiving an initial sustained inflation of 25 cmH_2_O for 15 s in the delivery room, aims to reduce BPD or death at 36 weeks postmenstrual age (35).

**Figure 5:**
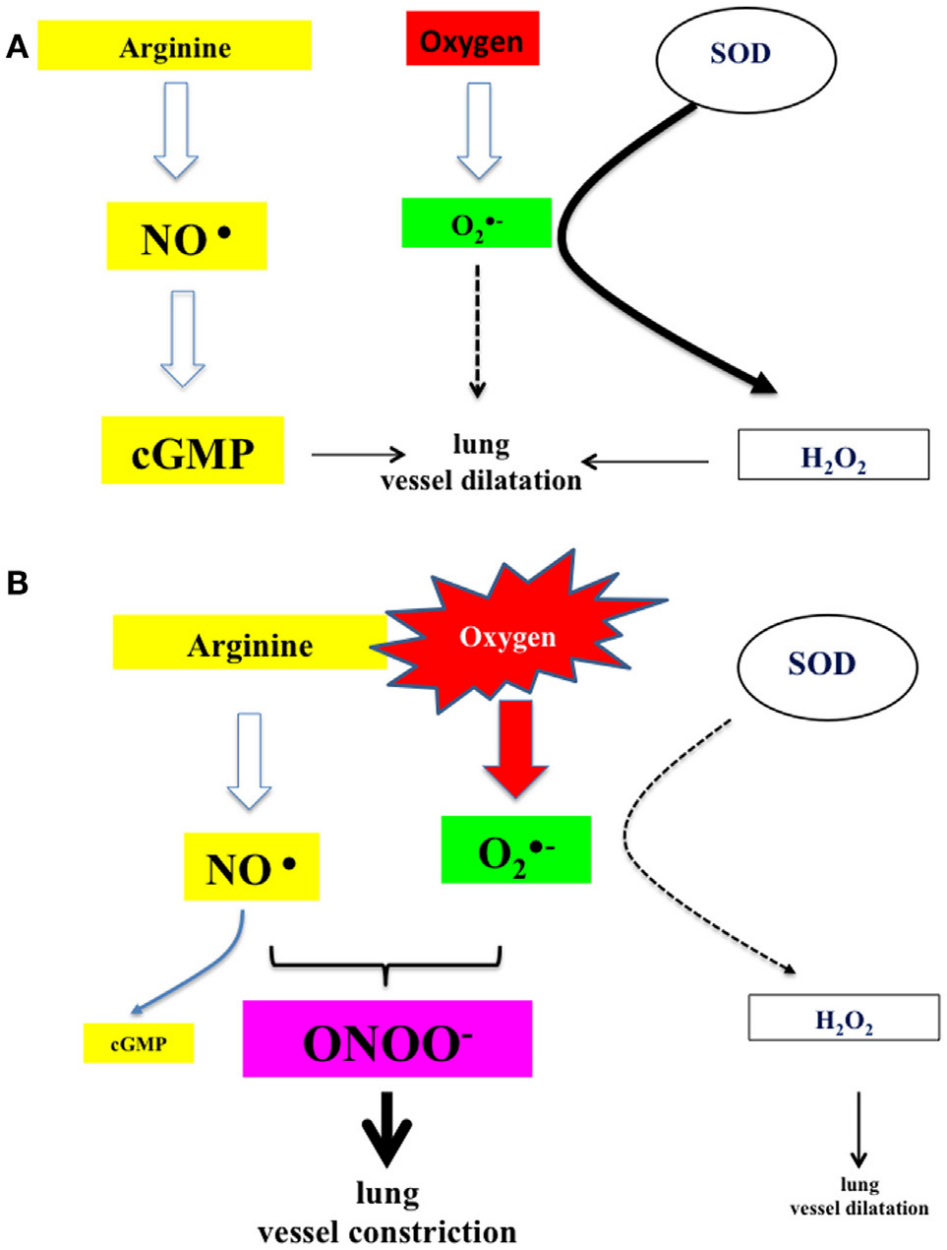
**(A)** Under normal circumstances, nitric oxide produced by the action of NO synthase activates guanylate cyclase, which catalyzes the formation of cGMP and subsequently lung vessel vasodilatation. In addition, superoxide anion, derived from air-borne oxygen by the action of superoxide dismutases, is catalyzed to hydrogen peroxide, which acts also as lung vessel vasodilator. **(B)** In the presence of oxygen in excess (resuscitation and ventilation), anion superoxide will sequester nitric oxide and produce highly reactive peroxynitrite. The amount of available cGMP is reduced and also the production of hydrogen peroxide. Under these circumstances, there is a potent tendency toward vasoconstriction (17, 25, 27).

Oxygen content of the inspiratory gas admixture decisively influences lung vascular tone and is considered one of the main factors causing vasodilatation in the fetal to neonatal transition (36). Traditionally, high oxygen inspiratory fractions have been recommended to enhance newly born infants postnatal adaptation, especially in asphyxiated term babies but also in preterm. However, experimental and clinical studies have shown that fetal to neonatal transition and oxygen supplementation cause an increased production of oxygen free radicals, especially anion superoxide (37). Under normal circumstances, exposure to a brief period of hyperoxia triggers the expression of SODs, which dismutate anion superoxide to hydrogen peroxide (Figure 5). H_2_O_2_ has a vasodilatation effect upon the lung vasculature, thus reducing vascular resistance and increasing pulmonary blood flow. However, in the presence of an excess of oxygen, non-dismutated anion superoxide may cause vasoconstriction by reducing the bioavailability of nitric oxide through the blocking the activity of eNOS and favoring the formation of the highly toxic peroxynitrite; moreover, in smooth muscle cells, free radicals inactivate soluble guanyl cyclase and activate phosphodiesterase P5, resulting in decreased levels of cyclic guanidine monophosphate. Altogether, these mechanisms lead to pulmonary vasoconstriction (Figure 5) (38, 39).

## Oxygenation before and after Birth

Alveolar–capillary gas exchange requires lung fluid resorption, lung expansion, and the establishment of a functional residual capacity before reaching a stable arterial partial pressure of oxygen (p_a_O_2_). In a term newborn infant, several minutes are needed after spontaneous breathing is initiated to complete this process. The evaluation of the metabolic and oxygenation status of the fetus and its correlation with immediate postnatal adaptation has been attempted using different approaches. Experimental studies in sheep models have shown that there is a good correlation between fetal scalp and preductal p_a_O_2_; however, there are physiological artifacts in the technique itself, such as aerobic contamination that may cause inaccurate results. Fetal pulse oximetry (FSpO_2_) in sheep has revealed that oxygen saturation before birth in the term fetus is stable around 50–70%. However, there is a “critical threshold” with FSpO_2_ <30% for more than 5–10 min. Prolonged low FSpO_2_ has been associated with low fetal arterial pH and lactic acid content (40). However, observational clinical studies have shown that both scalp pH and fetal pulse oximetry monitoring have not provided conclusive results and have not reduced the rate of cesarean section (41, 42).

After birth, the rate at which fetal SpO_2_ changes into newborn values is more gradual than previously believed. Newborn SpO_2_ levels do not reach stable values >90% until 5–15 min after birth elapse. This collides with the validity of pink color as one of the items valuated in the 1-min Apgar score. Based only on clinical inspection, there is a great variability in assessing newborn’s peripheral or even central color. Pulse oximetry has provided delivery room caregivers with an objective measurement of infant’s saturation and heart rate, which allows taking therapeutic initiatives as to how to stabilize each individual patient. Therefore, the use of pulse oximetry in the delivery room, especially for stabilization of preterm infants, is warranted (43, 44). However, in the recent ILCOR 2015 guidelines, there is a weak recommendation to use when available EKG instead of pulse oximetry to assess heart rate in the delivery room. EKG is meant to be more rapid and accurate; however, the evidence is indirect and imprecise, and therefore, this recommendation is considered weak and of very low quality (45).

The preliminary information regarding evolving SpO_2_ in the fetal to neonatal transition has been summarized by Finer and Leone in a recent review (46). In healthy term babies, pulse oximetry values retrieved in different observational studies were ≈60% at 1 min, 70% at 2min, 82% at 5 min, and 90% at 10 min. Moreover, pre- and postductal values differed in ~5–10% during the first minutes of life, and babies born by C-section had lower SpO_2_ values and needed longer time to achieve stable SpO_2_ >85% (46).

Dawson et al. developed an arterial oxygen saturation nomogram, aggregating three data sets from the Royal Women Hospital (Melbourne) and University and Polytechnic Hospital La Fe (Valencia). Data sets included preductal SpO_2_ retrieved every 2 s in healthy term and preterm infants during the first minutes after birth. Thereafter, data were mathematically treated to put up centile curves (47). Eligible infants had not received any intervention during stabilization period, such as supplemental oxygen or positive pressure ventilation, or had any congenital anomaly that could alter postnatal oxygenation. Of the total number of babies, 8% were preterm <32 weeks gestation, 26% 32–34 weeks gestation, and 66% term. The median to attain reliable SpO_2_ readings was 65 s (IC 5–95%: 58–85 s). Of note, there were significant differences between term and preterm infants along the first minutes after birth. Hence, while the median of term infants reached SpO_2_ >85% at 4 min after birth, preterm infants needed 7–8 min. Moreover, 10 min after birth, term babies were stable at 97% SpO_2_ and preterm at 94% (*p* < 0.001). In addition, babies born by vaginal delivery achieved stable saturations around 90% at 4–5 min, while babies born by C-section needed 6 min (47). There is an inverse correlation between gestational age and time to achieve stable SpO_2_ >85%, and therefore, the mort preterm infants will need longer time before they acquire a stable oxygenation level. Achievement of a SpO_2_ plateau after birth apparently correlates to time to eliminate fluid from the respiratory airways, recruit alveoli and expand the lung, and establish a functional residual capacity during expiration.

## Initial FiO_2_ in Preterm Infants

The 2015 ILCOR guidelines recommend that preterm infants should be initially stabilized using 0.21–0.30 oxygen inspiratory fraction (FiO_2_), aiming to avoid hyper- or hypoxemia (45). Studies performed in the last decade have shown that the use of 0.21 as initial FiO_2_ was insufficient to overcome hypoxemia and bradycardia in the first minutes after birth, while the use of higher oxygen concentrations (FiO_2_ = 0.9–1.0) caused hyperoxemia and oxidative stress. Wang et al. compared the use of 0.21 versus 1.0 as initial FiO_2_ in preterm infants with gestational age ≤28 weeks gestation. After 2 min, 30% of the patients were switched to 1.0 FiO_2_ because of persistent bradycardia, and at 3 min, the remaining 60% needed also an FiO_2_ of 1.0 because they did not achieve targeted SpO_2_ of 70% at 3 min (48). Remarkably, Vento et al. showed that the use of high initial FiO_2_’s of 0.90 caused hyperoxemia, oxidative stress, and an increased incidence of bronchopulmonary dysplasia (49). Kapadia et al. (50) randomized preterm infants into two different strategies. A group received an initial FiO_2_ of 1.0 and the other 0.21, and both were titrated according to SpO_2_ readings to achieve pre-established oxygenation targets. Results were coincidental with the study by Vento et al. (49). Hence, this study found an increased oxidative stress in the first hours after birth, more need of oxygen, more days on mechanical ventilation, and more BPD defined as the need for oxygen at 36 weeks postmenstrual age in the group that received 100% oxygen as initial gas admixture (50). In the interim, intermediate inspiratory fractions ranging from 0.3 to 0.5 have become generalized without strong evidence-based support. In a recent updated review and meta-analysis, 10 randomized studies including 321 infants who had received low (0.21–0.30) and 356 who received high (0.60–1.0) initial FiO_2_ were compared for relevant outcomes, such as mortality, bronchopulmonary dysplasia, and intraventricular hemorrhage (51). Of note, results from these meta-analysis showed that babies initially resuscitated with lower FiO_2_ approached almost significance for the outcome of reduced mortality [0.62 (95% CI: 0.37–1.04)]. Furthermore, there was no significant association for bronchopulmonary dysplasia or intraventricular hemorrhage when comparing low and high FiO_2_ (51). This information has been questioned by recent reports from two studies performed in Australia and Canada, respectively. Oei et al. (52) at the 2015 Pediatric Academic Societies Annual Meeting (USA) presented results of a large randomized controlled study completed to date, which examined the effects of resuscitation with an initial FiO_2_ of 0.21 versus 1.0 in 289 preterm infants <32 weeks gestation. Target SpO_2_ was set at 65–95% up to 5 min and 85–95% until admission to the NICU. The most relevant finding was a mortality of 16.2% in the subgroup of babies <29 weeks gestation in the 0.21 group versus 6% in the 1.0 group (RR = 3.18; 1.41–7.19; *p* = 0.013). This difference, although statistically marginal, emphasized the urgent need for larger randomized controlled trials to examine this question (52). Recently, the Canadian Neonatal Network published a retrospective cohort study comparing infant’s ≤27 weeks gestation before and after 2006 when the policy regarding initial FiO_2_ for preterm in the DR was changed from 100 to <100% oxygen and titration according to SpO_2_ (53). Adjusted OR (AOR) for the primary outcome of severe neurologic injury or death was higher in the lower oxygen group (AOR 1.36; 95% CI: 1.11–1.66) and in those resuscitated with RA (AOR 1.33; 95% CI: 1.04–1.69), when compared with 100% oxygen. Of note, researchers did not have data about oxygen exposure for each individual infant, and therefore, it could be misleading to attribute specific outcomes to the initial FiO_2_ used at delivery. However, the investigators cautioned that a policy of initial stabilization with lower oxygen could be linked to a higher risk of severe neurologic injury or death in very preterm infants compared to starting with 100% oxygen (53).

To define the best practice for the initial oxygen concentration for the transition and resuscitation of the very preterm infant requires the completion of additional well-designed and adequately powered randomized trials, such as the PRESOX (ClinicalTrials.gov Identifier: NCT01773746), and the Torpido 2 study that aims to examine outcomes of babies below 29 weeks gestation after initiation of resuscitation with either FiO_2_ 0.21 or 0.6. Hopefully, in the coming years, when both these studies are completed, we will have an answer to these burning questions.

## Conclusion

Oxygen is the most widely employed drug in Neonatology. Prematurity is associated with an immature respiratory and AO defense systems. Therefore, preterm infants frequently need oxygen supplementation to effectively transit from fetal to neonatal environment but develop oxygen free radical-associated conditions.

In recent years, experimental and clinical research studies have aimed to define the best approach to avoid damage caused by hyper- or hypoxia. Pursuing this objective a nomogram for oxygen saturation in the first minutes of life has been developed. Adjusting FiO_2_ to keep babies within the normality range has been proposed. However, there are still many unanswered questions: (i) which is the best starting FiO_2_? (ii) which is the best monitoring and titration practice to avoid hyper-or-hypoxia? (iii) which are the most appropriate SpO_2_ targets for very preterm infants? (iv) should all gestational ages be approached with the same initial FiO_2_? and (v) what should be done until the first reliable SpO_2_ reading is available?

Completion of two randomized controlled multicenter trials will hopefully provide us with the clues for an optimal use of oxygen in the fetal to neonatal transition.

## Author Contributions

All the authors included have contributed with their research work, review of the literature, drawing of the figures, and accepted the final version of the manuscript.

## Conflict of Interest Statement

The authors declare that the research was conducted in the absence of any commercial or financial relationships that could be construed as a potential conflict of interest.
